# Food insecurity and food pantry use among transgender and gender non-conforming people in the Southeast United States

**DOI:** 10.1186/s12889-020-08684-8

**Published:** 2020-04-29

**Authors:** Jennifer Russomanno, Jennifer M. Jabson Tree

**Affiliations:** The University of Tennessee Department of Public Health, 1924 Alcoa Highway, Knoxville, TN 37920 USA

**Keywords:** Transgender, Food insecurity, Health disparities, Food pantries, LGBTQ

## Abstract

**Background:**

Transgender and gender non-conforming (TGNC) people face high rates of poverty, joblessness, and homelessness, rendering this population vulnerable to experiencing food insecurity. Yet, there is almost no empirical evidence concerning food insecurity and the use of local and federal food assistance resources in the TGNC community. Food insecurity, the use of local and Federal food assistance resources, and associations with gender-related minority stressors and resilience using the Gender Minority Stress and Resilience (GMSR) scale among TGNC individuals living in the Southeast United States (U.S.) were documented in this study.

**Methods:**

A cross-sectional online survey was conducted with TGNC people living in the Southeast U.S. Participants were recruited via targeted Facebook advertisements.

**Results:**

In total, 105 TGNC people completed the survey; 79% of survey participants experienced food insecurity, 19% utilized Federal, and 22% utilized local food assistance resources. High levels of minority stress and community resilience were reported. The GMSR resilience scale Pride (aOR = 1.09, 95% CI 1.00–1.19, *p* = .04) was significantly associated with the use of local food pantries, but minority stressors were not. No significant associations were found between GMSR and food security.

**Conclusion:**

TGNC people living in the Southeast U.S. experienced food insecurity, unstable housing, low wages, and social stigma that were a barrier to using emergency food resources. Multi-level public health solutions that address discriminatory legislative policies and create linkages between TGNC people and local and federal food assistance are required to address issues of food insecurity in the TGNC population.

## Background

In the United States (U.S.), 1 in 9 people (11.1%) is food insecure [1]. Food security is defined as access by all people at all times to enough food for an active, healthy life and, at minimum, includes the availability of nutritionally adequate and safe foods, and the assured ability to acquire food in a socially acceptable way [2]. People who are able to meet these standards on a daily basis are considered food secure, and those that cannot are considered food insecure [2]. Federal organizations name food insecurity as a pressing public health challenge that contributes to hunger, obesity, chronic disease, and poor overall health [3–6].

Food insecurity disproportionately affects certain groups of people, including those living in poverty, people who are under or unemployed, and the homeless [3, 6, 7]. Transgender and gender non-conforming (TGNC) people (individuals whose gender identity is different from their sex assigned at birth) are a diverse group of people who experience some of the highest risks for food insecurity. Based on the 2015 U.S. Transgender Survey (USTS), [8] TGNC people are 4 times more likely to have incomes below $10,000/year, are 3 times more likely to be unemployed, and 2.5 times more likely to experience homelessness in their lifetimes, compared to cisgender (gender identity is concurrent with sex assigned at birth) counterparts [8].

TGNC people also face the burden of minority stressors in the form of discrimination, stigma, and marginalization as a result of their gender identities. Minority stress is a chronic, cumulative, and institutional source of stress built into how organizations and societies function and exists beyond the control of the individual or subgroup that it targets [9]. Minority stress affects TGNC people’s internal perceptions of themselves, their relationships with peers, family members and community members, their integration and acceptance into community groups, clubs or religious institutions, and their rights as dictated by governmental laws and legislation [9–11].

Although TGNC people are exposed to minority stressors, this group is also resilient. Meyer [10] referred to this resilience as community resilience, or *minority coping*. Community resilience is a sense that individuals can overcome life challenges and obstacles with the assistance of close community networks and support. For TGNC people with strong community or social support, community resilience may be protective against minority stress [10]. Multi-level minority stressors and community resilience may contribute to or protect against the negative consequences of food insecurity in the TGNC population, however, these associations have not yet been empirically explored and documented.

Where a person lives also influences their experiences with and risk for food insecurity, minority stress, and community resilience. TGNC people living in states with high rates of food insecurity and sociopolitical contexts that produce discrimination and stigma, may be at especially high risk for food insecurity. Of the 12 Southeast states (Alabama, Arkansas, Florida, Georgia, Kentucky, Louisiana, Mississippi, North Carolina, South Carolina, Tennessee, Virginia, West Virginia), 9 have food insecurity rates above the national average [12]. Additionally, the Southeast states, have, on average, very high levels of social stigma toward Lesbian, Gay, Bisexual & Transgender (LGBT) people, [11] evidenced by the absence of employment or non-discrimination laws that protect this population [11]. High levels of social stigma combined with non-protective laws decrease the safety, economic stability, and acceptance of the estimated 380,000 TGNC people living in the Southeast U.S. [11, 13]

In the general population, local food assistance programs, such as food pantries, are valuable resources to alleviate food insecurity [14]. A majority of U.S. food pantries (67%) are run by faith-based institutions, [15] which could pose a great threat to food insecure TGNC people. State Religious Freedom Restoration Acts (RFRA), or “religious freedom laws” allow institutions, including food pantries, to deny services to select community members based on religious beliefs [16]. These laws allow food pantries to deny TGNC people support and thereby further jeopardize food access to this population [17].

### Study purpose

The purpose of this study was two-fold. First, we aimed to describe TGNC peoples’ experiences with food insecurity, including their use of Federal and local food assistance resources. Second, we sought to investigate possible associations between gender-related minority stressors and community resilience and the experience of food insecurity and use of local food assistance resources among TGNC people in the Southeast U.S.

## Methods

The University of Tennessee Institutional Review Board approved all study procedures (UTK IRB-18-04907-XP).

### Pilot test

To ensure survey quality and that questions were appropriate and sensitive to the priority population, the survey was pilot tested with a person who self-identified as transgender male, and who resided in Tennessee. The pilot tester provided positive feedback about the survey questions and did not suggest modifications to the survey protocol. The survey was not modified after the pilot test, prior to public dissemination.

### Recruitment

From January to February 2019, TGNC people living in the Southeast U.S. were recruited online via targeted Facebook advertisements to complete an online survey. This recruitment approach is an evidence-based method documented to successfully recruit stigmatized groups into research projects [18–21].

### Eligibility

Eligibility was determined with a 4-item online eligibility questionnaire. Determining eligibility required potential survey participants to: (1) agree to participate via informed consent, (2) live in 1 of the 12 Southeast U.S. states, (3) be over age 18, and (4) self-identify as TGNC. Responses were required for each item before it was possible to advance to the full survey instrument. Upon clicking on the survey link embedded in the Facebook advertisement, potential respondents were directed to an online informed consent form with response options of “agree to participate” or “decline to participate.” Respondents who selected “decline to participate” were considered ineligible to participate, and were directed to the end of the survey, thanking them for their time. Respondents who selected “agree to participate” were directed to the remaining items on the eligibility questionnaire.

Survey participation was voluntary and confidential. Participants could voluntarily provide a name and email address to be entered to win 1 of 4 randomly-selected $50 electronic gift cards. Names and email addresses were not associated with survey responses.

#### Participation

Seven hundred and forty-two people clicked the survey link. Of those, 166 (22.4%) consented to participate. Nineteen participants (11.4%) either did not meet eligibility criteria (*n* = 16) or left the survey prior to the first question of the full survey (*n* = 3).

### Measures

The cross-sectional survey was conducted online via Qualtrics and measured: (1) food insecurity; (2) use of local and Federal food assistance resources; (3) gender-related stress and resilience; and (4) demographic characteristics. All “prefer not to answer” responses to any survey item were coded as missing.

#### Food insecurity

Food insecurity was assessed using the USDA 6-item Short Form Food Security Module [22]. This measure identifies food insecure households [22] with Cronbach’s alpha (α) ranging from 0.74 to 0.93 [23]. USDA-recommended scoring guidelines [2] were used to calculate participant food security scores. Overall scores were summed, with a total possible score of 0–6. Final participant scores were dichotomized: food secure (raw score 0–1) and food insecure (raw score 2–6) [22].

#### Local and federal food assistance programs

Two survey items were used to assess participants’ experiences with the Federal Supplemental Nutrition Assistance Program (SNAP), and 6 items assessed participants’ experiences with local food pantries (Table 1). These items were designed by the research team specifically for this survey. Skip progressions were used for questions regarding federal food assistance and use of local food assistance programs. Regarding Federal food assistance, all participants were asked whether they had received SNAP benefits in the past 12 months. If a participant indicated they had received benefits during this period, they were directed to an additional follow-up question assessing their average monthly SNAP allowance. Regarding local food assistance programs, all participants were asked whether they had use local food assistance programs in the past 12 months. If a participant indicated they were not currently using food pantries, they were directed to a follow-up question regarding why they currently did not utilize these resources. All participants were asked the remaining 4 questions regarding local food assistance resources capturing organizing agencies of their local pantries and assessing the welcoming nature of pantries to TGNC people.

**Table 1:** Local and Federal food assistance program survey questions

Question	Potential Responses
1. Do you currently, or have you in the past 12 months, received assistance through the Supplemental Nutrition Assistance Program (SNAP) (formerly known as food stamps)? [all participants]	1. Yes, I/We currently receive SNAP assistance
	2. Yes, I/We have received SNAP assistance in the past 12 months, but do not currently receive assistance
	3. No, I/We have not received SNAP assistance in the past 12 months
	4. Prefer not to answer
2. On average, how much in SNAP assistance did/do you receive monthly? [participants who responded Yes in Q1]	1. Less than $50
	2. $50 - $99
	3. $100 - $149
	4. $150 - $199
	5. $200 or more
3. Do you currently, or have you in the past 12 months, use local food assistance programs such as food pantries? [all participants]	1. Yes, I/We currently use them
	2. Yes, I/We have used them in the past 12 months, but do not currently
	3. No, I/We do not use them and have not used them in the past 12 months
	4. Prefer not to answer
4. Why do you not currently use local food pantries? (select all that apply) [participants who responded no current food pantry use in Q3]	1. I do not need to use these resources at this time
	2. I do not feel these resources are meant for me
	3. I do not feel comfortable using these resources
	4. I do not have transportation to these resources
	5. I do not feel welcome at these resources
	6. I did not know these resources existed
	7. None of these resources are available to me in my community
	8. Other, please describe
	9. Prefer not to answer
5. Who is the organizer of your local food pantry? (if more than one pantry exists in your community, select all that apply) [all participants]	1. Church or faith-based organization
	2. Charitable, non-profit organization (such as Second Harvest)
	3. A local college or university
	4. I am not aware of any food pantries in my community
	5. I do not know who organizes my local food pantry
	6. Prefer not to answer
6. Overall, how welcoming do you feel your local food pantry is to transgender or gender non-conforming people? [all participants]	1. Extremely welcoming
	2. Somewhat welcoming
	3. Neither welcoming nor unwelcoming
	4. Somewhat unwelcoming
	5. Extremely unwelcoming
7. In your own words, please describe how your local food pantry is **welcoming** to transgender or gender non-conforming people. [all participants]	Open ended
8. In your own words, please describe how your local food pantry is **unwelcoming** to transgender or gender non-conforming people. [all participants]	Open ended

#### Gender-related stress and resilience

Gender-related stress and resilience were assessed using the Gender Minority Stress and Resilience (GMSR) scale [24]. The Cronbach’s alphas reported in previous studies using the GMSR scale ranged from 0.61 *(gender-related discrimination*) to 0.93 (*negative expectations for the future*) [24, 25]. GMSR scoring guidelines [24] were used to calculate participant scores within each subscale. Higher scores indicated a higher minority stress and/or resilience depending on subscale.

#### Demographics

Demographic variables included: *state of residence*, *gender identity,* [26] *age,* [27] *geographic location* (urban, suburban, rural), *marital status,* [27] *race,* [27] *ethnicity,* [27] *education,* [27] *employment status,* [27] *household income,* [27] *housing stability,* [28] and *number of children in the household* [27].

### Statistical analyses

#### Food insecurity and local and federal food assistance

Summary statistics were calculated for food insecurity and receipt of SNAP benefits and use of local food pantries. Open-ended questions concerning experiences with local food assistance resources were analyzed qualitatively by categorizing and sorting the responses into overarching themes.

#### Gender-related minority stress

Mean GMSR subscale scores were calculated.

#### Demographics

Frequencies and percentages for all demographic variables were calculated.

#### Associations between GMSR and food insecurity/local and federal food assistance

Logistic regression models were calculated with adjustment for demographic characteristics, to test for associations between GMSR subscales and (1) food pantry usage and (2) food security. All adjustment variables were selected based on associations in bivariate analyses. Per published guidelines [29], variables that were significant at < 0.15 in the bivariate analyses were included as adjustment variables in the multivariable regression models. The six regression models calculated were: (1) food pantry usage regressed on the 7 full-sample GMSR subscales; (2 & 3) food pantry usage regressed on the 2 partial-sample GMSR subscales (*negative expectations for the future* and *nondisclosure*) for the “gender history” and “gender identity” participant subgroups; (4) food security regressed on the 7 full-sample GMSR subscales; and (5 & 6) food security regressed on the 2 partial-sample GMSR subscales for the “gender history” and “gender identity” participant subgroups.

Logistic regression models testing the associations between GMSR and food security and use of food pantries were calculated as nested models, with demographic covariates included in Block 1, and GMSR subscales in Block 2.

#### Missing data

As recommended [30, 31], respondents were excluded from analyses if they were missing more than 20% of survey items. In our tests of missingness according to recommended guidelines [31, 32], there were no obvious patterns of missingness within excluded cases χ ^2^ (18, *N* = 147) 17.11, *p* = .52. Therefore, cases were considered missing completely at random (MCAR). According to Peng [31], MCAR assumes the “probability of a response depends on neither the observed nor the missing value that could have been collected or recorded” among cases with 20% missing data. Under conditions where data are MCAR, complete removal of cases with 20% or more missing data will not produce biased estimates to the remaining data [31]. For this project, 42 MCAR cases were excluded, leaving 105 cases in the final analyses.

Respondents who were missing fewer than 20% of survey items had item-level missing data handled by multiple imputation (MI) or mean substitution based on the percent of data missing [30, 31, 33]. In the remaining 105 cases, there were varying levels of missing data at the item level ranging from 17.1% (food security) to 0% (state of residence; geographic location; number of children in household). Given the level of missingness in food security (17.1%), MI was conducted. The rate of missing information in the food security variable did not exceed 50%, therefore, based on published guidelines, 5 imputations were used in the MI model [34, 35]. The predictive variables used to impute food security in the model were receipt of SNAP benefits and use of local food pantries. These two variables were chosen as predictor variables based on their association with food insecurity in the general population [14, 36]. For variables with low level missing data (< 4%), mean substitution was utilized [33]. Previous studies suggest that mean substitution is comparable to more complex methods at when low levels of missingness are present [31, 33]. All analyses were conducted using IBM SPSS Statistics Version 25 [37].

#### Sample size and power analysis

Post hoc power analyses were calculated [38] using the GMSR scale and applying the widest 95% CI and widest standard deviation (95% CI: 13.8–17.2; sd = 8.81). The statistical analysis had 80% power to detect associations if was associations were present (*n* = 105).

## Results

### Participants

**Fig. 1:**
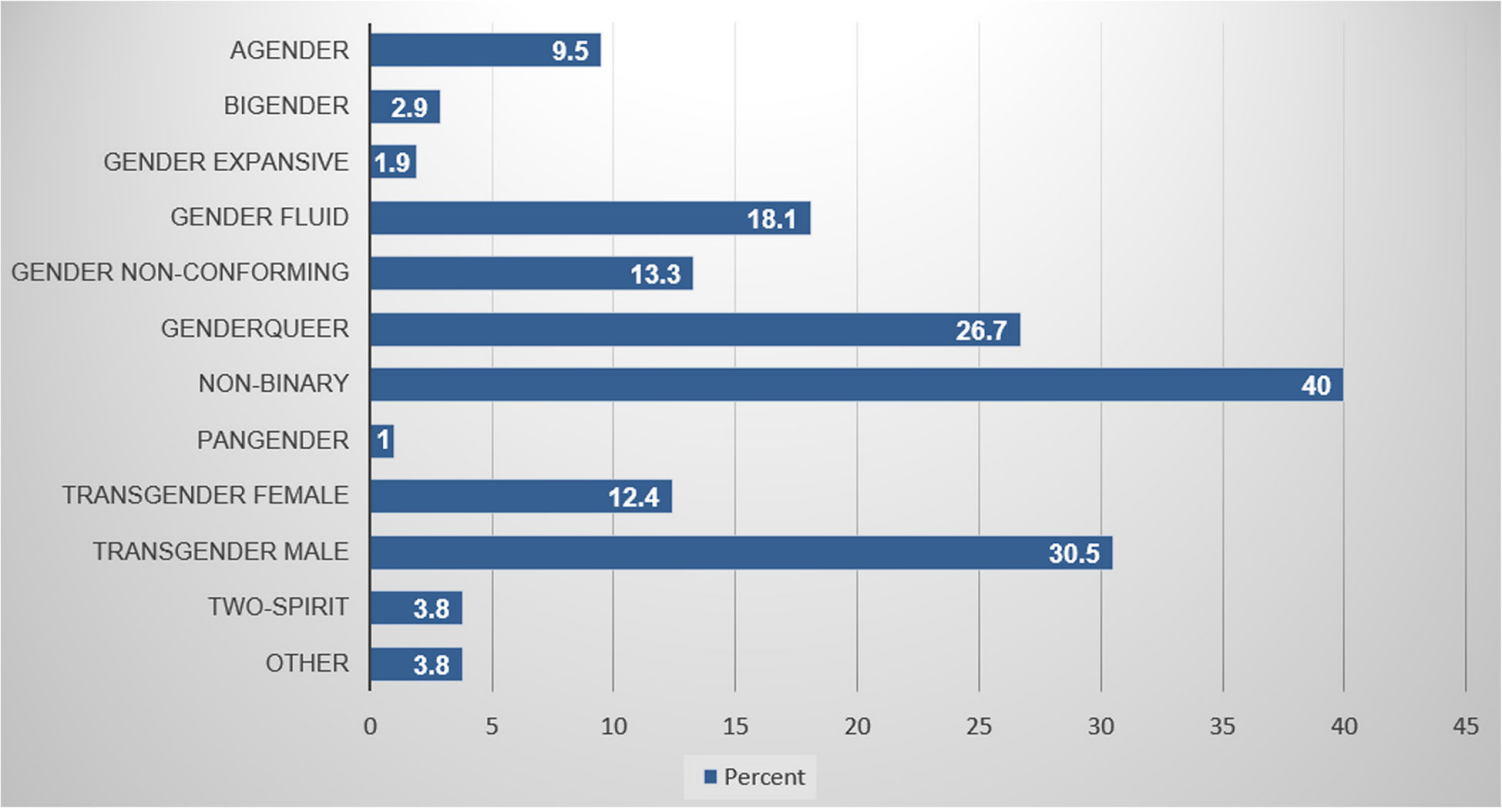
Survey participant gender identities

Within our study sample, we had at least one participant from each of the 12 Southeastern U.S. states. Most participants were White (85.7%), non-Hispanic (94.3%), employed or self-employed (68.6%), and had at least some college education (80%). Of the participants that did not identify as White (*n* = 15), 6.7% (*n* = 7) identified as mixed race, 3.8% (*n* = 4) identified as Black or African American, 1.9% (*n* = 2) identified as “other,” 1% (*n* = 1) identified as American Indian, and 1% (*n* = 1) identified as Asian. The mean age of our study sample was 27.4 years (sd = 8.6). Participants identified across a range of gender identities; the majority identified as non-binary (40%), transgender male (30.5%), and/or genderqueer (26.7%) (Fig. 1).

### Food insecurity

Table 2 contains a summary of the pooled results of differences in food security and food pantry by sample demographic characteristics. Food insecurity was identified among 79% of participants (*n* = 83). Among those who were food insecure, 20% (*n* = 17) reported low food security, and 80% (*n* = 66) reported very low food security. The remaining 21% of participants (*n* = 22) were food secure. Among those who were food secure, 68.2% (*n* = 15) reported high food security and 31.8% (*n* = 7) reported marginal food security.

**Table 2:**
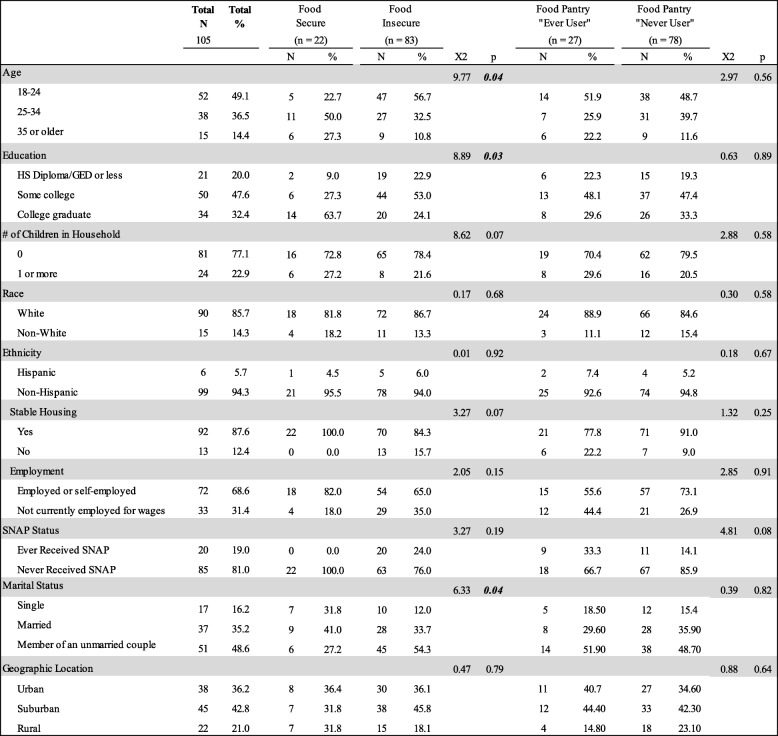
Food security status and food pantry usage by demographic characteristics (*n* = 105)

### Food assistance resources

The majority of participants reported never receiving SNAP benefits (*n* = 85, 81%) and never using local food pantries (*n* = 78, 74.3%). Participants reported not using food pantries for multiple reasons, ranging from feeling like food pantries were not meant for them (*n* = 43, 41%) to not having adequate transportation (*n* = 12, 11.4%). Participants reported a variety of food pantry organizers, including faith-based organizations (*n* = 53, 50.5%), non-profit organizations (*n* = 29, 27.6%) and local colleges (*n* = 9, 8.6%).

Participants reported feeling unwelcome at local food pantries due to their TGNC status, particularly at pantries operated by faith-based organizations (Table 3). One participant stated, “People are required to sit through a church service, and the pastor has spoken against homosexuality, which made me uncomfortable.” Another recalled, “It is Christian based and that tends not to go well for us here.” Participants also suggested that food pantries may be unwelcoming due to the socio-conservative climate of the Southeast U.S. One participant stated, “I’m in the Bible Belt, they say we do not exist or are mentally challenged.”

**Table 3:** Open-ended comments regarding the welcoming nature of food pantries

*In your own words, please describe how your local food pantry is****welcoming****to transgender or gender non-conforming people.*	
• They are very welcoming. Just very nice to everyone.	
• I believe it’s more of a “what they don’t know won’t hurt them” situation; if they don’t know that I/someone am/is trans or gender-non-conforming, then there isn’t a problem.	
• I imagine the one at the college I work at is much more welcoming than the religious pantry located nearest me.	
• It is run by my college, which is accepting to the LGBT community.	
• They did not care either way or didn’t notice.	
• We don’t know; my girlfriend and I are both pre-op and stealth (I present female and she presents male in public).	
• Food Not Bombs is very welcoming	
• Second Harvest is supported by the UU church, and they support TGNC people.	
• The food pantry is at my local church (ORUUC) but offers this to anyone in need in the local community. I felt welcoming and like they treated me like they would anyone else that came to them for assistance.	
*In your own words, please describe how your local food pantry is****unwelcoming****to transgender or gender non-conforming people.*	
• Everybody stares, whispers of “what is that”, “she’s just confused”	
• We aren’t the normal racist “Christians” that seem to be the majority here.	
• Hateful angry passive aggressive or just aggressive. Refusing prejudice and making remarks towards the community Spectrum. Often times I met with threats of violence or hateful looks and am treated unfairly among places like the food pantry or food stamp office to a point to where paperwork has been manipulated, dodged, thrown away, or edited	
• We are surrounded by hate and transphobes	
• People are required to sit through a church service, and the pastor has spoken against homosexuality, which made me uncomfortable.	
• Tells us we are going to hell or there’s only two genders and go seek medical assistant	
• Transgender people not allowed	
• The area I live in is very bigoted, and the community harbors hate for lgbtqia folks.	
• I’m in the Bible Belt, they say we do not exist or are mentally challenged	
• It is Christian based and that tends not to go well for us here.	
• Tennessee resents people like us. That’s fact.	
• It’s in a church and not one of the ones I know tends to be lgbt friendly	
• FISH pantries may be staffed by trans/homophobic volunteers	
• I haven’t experienced being unwelcome at my local food pantry but I feel unwelcome by most religious based organizations and many organizations in general in this area unless I know they’re LGBTQ friendly and welcoming.	

### Gender-related minority stressors and community resilience

Table 4 provides a summary of participants’ mean GMSR scores, including alphas from our sample. The highest reported scores were *gender-related rejection* (M = 4.07, sd = 1.48) and *non-affirmation of gender identity* (M = 18.83, sd = 5.77). In response to the question, “Do you currently live in your affirmed gender all or almost all of the time,” 74 participants (70.5%) stated they lived in their affirmed gender all or almost all of the time and were presented with the terminology of “gender history” in the subscales of *negative expectations for the future* and *nondisclosure*. Thirty-one participants (29.5%) stated they did not live in their affirmed gender all or almost all of the time and were presented with the terminology of “gender identity” in the aforementioned subscales.

**Table 4:** Gender minority stress and resilience (GMSR) measure summary statistics

Subscale	N	Min	Max	Mean (*sd*)	α
Gender-related discrimination	105	0	5	2.77 (*1.45*)	.60
Gender-related rejection	105	0	6	4.07 (*1.48*)	.55
Gender-related victimization	105	0	6	2.54 (*1.93*)	.79
Non-affirmation of gender identity	105	0	24	18.83 (*5.77*)	.90
Internalized transphobia	105	0	32	15.51 (*8.81*)	.90
Pride	105	0	32	18.48 (*7.85*)	.88
Negative Expectations for the future (Gender history)	74	0	36	23.29 (*8.06*)	.89
Negative Expectations for the future (Gender identity)	31	0	36	28.57 (*5.92*)	.86
Nondisclosure (Gender history)	74	0	20	12.13 (*6.25*)	.88
Nondisclosure (Gender identity)	31	0	20	12.87 (*5.43*)	.83
Community connectedness	105	0	20	12.19 (*4.94*)	.83

### Associations between GMSR, food pantry usage, and food security

Food pantry usage was regressed on the GMSR subscales, with adjustment for receipt of SNAP benefits (Table 5). There was a positive association between the *Pride* subscale and the use of food pantries (aOR = 1.09, 95% CI 1.00–1.19, *p* = .04). Participants who were more self-assured in their gender identity were 9% more likely to use food pantries than those who were less-assured. No significant associations were found between any GMSR subscales and food security (see Supplemental Table 1).

**Table 5:** Binary logistic regression predicting likelihood of food pantry usage by full-sample GMSR subscale scores, when adjusting for SNAP status X^2^ (8, 105) = 13.13 *p* = .11

	aOR	95.0% C.I.		p
***Block 1***		**LL**	**UP**	
***SNAP status***				*0.03*
Never received SNAP	*ref*			
Ever received SNAP	3.64	1.13	11.67	0.03
***Block 2***				
***GMSR Subscales (full sample)***				
Gender-related discrimination	0.87	0.55	1.38	0.56
Gender-related rejection	0.81	0.52	1.26	0.35
Gender-related victimization	1.23	0.84	1.80	0.30
Non-affirmation of gender identity	0.98	0.88	1.08	0.65
Internalized transphobia	0.99	0.92	1.06	0.75
Pride	1.09	1.00	1.19	***0.04***
Community	0.91	0.81	1.02	0.10
**Constant**	2.06			0.63

## Discussion

In this study, we set out to document: (1) food insecurity experiences had by TGNC individuals living in the Southeast U.S., (2) the experiences had by TGNC people when utilizing Federal and local food assistance resources, and (3) how gender-related minority stressors and community resilience relate to food insecurity and the use of local food assistance resources.

A majority of survey participants experienced food insecurity (*n* = 83; 79%), and few participants utilized Federal (*n* = 20, 19%) and local (*n* = 27, 25.7%) food assistance resources. Participants had high levels of minority stress and community resilience. Minority stressors were not related to food insecurity or the use of local food pantries. However, community resilience was associated with local food pantry usage.

There is very little evidence about food insecurity in the TGNC population, and there are several gaps in what is known. The existing literature combines experiences of food insecurity among TGNC people with people who identify as gay, lesbian, and bisexual. This makes it impossible to understand the unique experiences of TGNC people. Additionally, the existing evidence does not use a rigorous measurement for food insecurity. For example, Brown and colleagues [39] found that 27% of LGBT adults experienced a time in the last year when they did not have enough money to feed themselves or their families. However, two methodological issues [39] limit what can be understood from Brown and colleagues about food insecurity in the TGNC population. First, only one of the four data sources used by Brown’s study assessed transgender inclusive gender identity, and gender identity and sexual orientation questions were combined in a single identity measure [39]. This is problematic because it is impossible to determine how many of the food insecure participants reported by Brown and colleagues identify as transgender, and therefore the proportion of TGNC people who experience food insecurity.

Second, the TGNC-inclusive data source in Brown and colleagues’ project used only a single-item to assess food security [39]. A single measure does not accurately assess the dynamic complexities and factors of food insecurity, made up of multiple factors including: access to food, whether food supplies are regular and consistent, and whether available food is healthy and filling [39]. We used the USDA approved 6-item assessment of food security in order to more fully inform the dynamic experience of food insecurity among TGNC people.

It is possible that the rates of food insecurity for TGNC people are likely to be much higher than what we identified with our project. From an economic perspective, our study produced findings similar to those published from the 2011 U.S. National Transgender Discrimination Survey (NTDS). The NTDS described structural, economic, and health challenges faced by transgender people in the U.S. For example, 14% of Southeast U.S. NTDS respondents had yearly incomes under $10,000, and 14% experienced unstable housing in the past year due to their gender identity/expression [40]. In our study, 19% of survey respondents reported yearly incomes of less than $10,000, and 12.7% experienced unstable housing within the past 2 years. Future studies should consider how under and unemployment as well as household instability are associated with food insecurity among the TGNC population, primarily in regions of the U.S. that do not have anti-discrimination housing or employment policies in place.

In their study assessing health outcomes of LGBT people in various regions of the U.S., Hasenbush and colleagues [11], found that a high percentage of LGBT people living in states without anti-discrimination protection laws had at least some college education. They concluded that LGBT people living in states without legislative protections (including all of the Southeast U.S. states) may seek advanced education to bolster their employment prospects in response to workplace discrimination that might be encountered in these areas [11].

Participants in our study may be similar to those who participated in the Hasenbush study. Despite low annual incomes reported by survey respondents, a majority (79.6%) had some higher level education. Of those that reported having at least some college education, 41.5% were college graduates. This is consonant with hypotheses that marginalized people living in states with high levels of social stigma seek higher educational attainment in an effort to combat potential discrimination in workplace settings.

Previous studies suggest that TGNC people experience high levels of harassment [41], employment discrimination [42], and community discrimination [42, 43] due to their TGNC status. Bradford and colleagues [42] assessed gender-related discrimination among transgender people in Virginia and found that 41% reported experiencing gender-related discrimination in one or more of the following areas: health care, employment, and housing. Results from our study are similar. Above-the-mean scores were observed in 3 of the GMSR subscales assessing minority stress and social stigma: gender-related discrimination, gender-related rejection and non-affirmation of gender identity, indicating that survey respondents were experiencing high levels of social stigma, rejection, and discrimination in their communities.

Results from our study indicated that personal pride in one’s TGNC identity was associated with greater likelihood of using local food pantries. It is possible that personal pride could be protective against minority stress and social stigma. In their qualitative study of transgender people of color residing in the Southeast U.S., Singh and McKleroy [44] found that participants with a higher sense of pride were better able to overcome barriers of transphobia and racism within their communities than those with a lower sense of pride. It is possible that TGNC people who are proud of their gender identity may be better equipped to overcome potential issues of discrimination or transphobia when securing food from local food pantries than those who may feel less proud of their gender identity.

### Limitations

This study has limitations. Participants were purposively recruited via convenience sampling through targeted Facebook advertisements. Additionally, a vast majority of survey participants were white, non-Hispanic. Thus, our results cannot be generalized past our sample’s demographic characteristics.

In total, 742 people clicked on the Facebook advertisement, but only 166 people actually consented to participate and moved forward to the eligibility screening. No data were collected from potential participants prior to consenting to the project, therefore it is not possible to determine why 166 of the 742 consented to participate and others did not. It is conceivable that individuals were initially curious about the project, but realized that they were not eligible, or were not interested in participation. It is possible, although not testable, that individuals who consented to participate are different in important ways from those who did consent to participation, and this limits the generalizability of this study.

## Conclusions

Several interrelated, multi-level, public health solutions are required to alleviate issues of food insecurity and food access in the TGNC population. Federally, national population-based surveys assessing food security in the general population should be required to capture gender identity so public health professionals can use the most rigorous epidemiological methods to accurately assess food insecurity within the TGNC population. Currently none of the health surveillance programs include TGNC inclusive measures of gender [45]. Structurally, Federal or State level legislation must be established to protect TGNC people from social stigma and discrimination in employment, housing, and healthcare. Federally, and within the Southeast U.S. states, laws do not guard against discrimination for TGNC individuals [11, 46]. RFRA laws actively allow institutions and employers to deny services and opportunities to TGNC people based on religious beliefs. Lack of legal protection and discriminatory laws jeopardize TGNC peoples’ employment opportunities, housing options, food security, and access to community food resources. Future research should consider exploring food insecurity and food assistance use by TGNC people on a national level, allowing for comparisons of TGNC people residing in states with and without anti-discrimination policies in place.

Given the majority of survey participants were food insecure, yet very few sought help from local food assistance resources, several community-wide solutions could also be implemented to ensure food insecure TGNC people have safe, affirming resources for food. One possible solution is for TGNC community organizations to partner with local food pantries to ensure a non-threatening, welcoming environment is being created for TGNC people in need of food assistance. In addition, local food pantries and TGNC community organizations could work together to provide “pop-up” food pantries in places that are easily accessible to TGNC people in need.

## Supplementary information

**Additional file 1: Table S1.** Binary logistic regression predicting likelihood of food insecurity by full-sample GMSR subscale scores, when adjusting for age, education, children, housing stability, and marital status X^2^ (8, 105) = 33.04 *p* = .005.

## Data Availability

The datasets used and/or analyzed during the study are available from the corresponding author on reasonable request.

## Abbreviations

GMSR: Gender Minority Stress and Resilience Scale
LGBT: Lesbian, Gay, Bisexual & Transgender
NTDS: 2011 National Transgender Discrimination Survey
RFRA: Religious Freedom Restoration Acts
SNAP: Supplemental Nutrition Assistance Program
TGNC: Transgender and Gender Non-conforming
U.S.: United States
USDA: United States Department of Agriculture
USTS: 2015 U.S. Transgender Survey
