# Supplementary material for: Food insecurity and food pantry use among transgender and gender non-conforming people in the Southeast United States
Source: BMC Public Health. 2020 Apr 29;20:590. doi: 10.1186/s12889-020-08684-8 (PMC7191729; doi:10.1186/s12889-020-08684-8)
Supplement: Supplementary file 1 — Additional file 1: Table S1. Binary logistic regression predicting likelihood of food insecurity by full-sample GMSR subscale scores, when adjusting for age, education, children, housing stability, and marital status X2 (8, 105) = 33.04 p = .005. [file 12889_2020_8684_MOESM1_ESM.docx]

**Supplementary Table 1.** Binary logistic regression predicting likelihood of food insecurity by full-sample GMSR subscale scores, when adjusting for age, education, children, housing stability, and marital status X^2^ (8, 105) = 33.04 p = .005*

|  | **aOR** | **95.0% C.I.** | | **p** |
| --- | --- | --- | --- | --- |
| ***Block 1*** |  | **LL** | **UP** |  |
| ***Age*** |  |  |  |  |
| 18-24 | *ref* |  |  |  |
| 25-34 | 1.35 | 0.09 | 19.99 | 0.82 |
| 35 or older | 1.33 | 0.97 | 18.25 | 0.83 |
| ***Education*** |  |  |  |  |
| High School Diploma/GED or less | *ref* |  |  |  |
| Some College | 0.02 | 0.00 | . | 0.99 |
| College Graduate | 0.01 | 0.00 | . | 0.99 |
| ***Number of Children in Household*** |  |  |  |  |
| 0 | *ref* |  |  |  |
| 1 or more | 1.19 | 0.20 | 7.36 | 0.86 |
| ***Stable Housing*** |  |  |  |  |
| Yes | *ref* |  |  |  |
| No | . | . | . | 1.00 |
| ***Marital Status*** |  |  |  |  |
| Single | *ref* |  |  |  |
| Married | 1.50 | 0.21 | 10.60 | 0.69 |
| Member of an Unmarried Couple | 5.26 | 0.77 | 36.02 | 0.09 |
| ***Block 2*** |  |  |  |  |
| ***GMSR Subscales (full sample)*** |  |  |  |  |
| Gender-related discrimination | 1.22 | 0.51 | 2.89 | 0.64 |
| Gender-related rejection | 0.84 | 0.45 | 1.58 | 0.59 |
| Gender-related victimization | 1.50 | 0.82 | 2.74 | 0.19 |
| Non-affirmation of gender identity | 1.12 | 0.99 | 1.27 | 0.08 |
| Internalized transphobia | 1.10 | 0.90 | 1.15 | 0.75 |
| Pride | 1.02 | 0.89 | 1.16 | 0.81 |
| Community | 1.05 | 0.87 | 1.27 | 0.60 |
| **Constant** | 1.86 |  |  | 1.00 |

**All results presented in this table are based on pooled analyses*
